# Management of Recurrent and Aggressive Non-Functioning Pituitary Adenomas

**DOI:** 10.3390/jcm14155203

**Published:** 2025-07-23

**Authors:** Nicole A. Hefner, Odelia Cooper

**Affiliations:** 1 Department of Medicine, Cedars-Sinai Medical Center, Los Angeles, CA 90048, USA; nicole.hefner@cshs.org; 2 Pituitary Center, Cedars-Sinai Medical Center, Los Angeles, CA 90048, USA

**Keywords:** nonfunctioning pituitary adenoma (NFPA), temozolomide, somatostatin receptor ligand (SRL), dopamine agonist, immune checkpoint inhibitor, vascular endothelial growth factor (VEGF) inhibitor, peptide receptor radionuclide therapy

## Abstract

When non-functioning pituitary adenomas (NFPAs) behave aggressively or recur after first-line surgical treatment, it can be challenging to decide whether and how to escalate therapy. Up to 47% of patients with residual tumor after transsphenoidal surgery will show disease recurrence or progression and may require an intervention. Repeat surgical resection can be attempted in select cases if the tumor is accessible; for the remainder of patients, non-surgical treatment options may need to be considered. Radiotherapy can control tumor growth in 75% of NFPAs, but confers increased risk of hypopituitarism and other disorders. Currently, there are no medical therapies approved for patients with recurrent or aggressive NFPA. However, several have been investigated, including temozolomide, somatostatin receptor ligands, dopamine agonists, immune checkpoint inhibitors, vascular endothelial growth factor inhibitors, and peptide receptor radionuclide therapy. We present a review of the available evidence to provide guidance for pituitary endocrinologists and neuro-oncologists when treating patients with recurrent or aggressive NFPA.

## 1. Introduction

Pituitary adenomas are generally benign, and many are identified incidentally on brain imaging conducted in patients with no overt signs or symptoms. The prevalence of clinically relevant pituitary adenomas is estimated at 830 cases per 1 million individuals [1]. Aggressive adenomas comprise 2% of resected pituitary adenomas, and pituitary carcinomas comprise <0.1% of all detected anterior pituitary tumors [1,2].

Non-functioning pituitary adenomas (NFPAs), which show no biochemical or clinical manifestations of hormone hypersecretion, constitute approximately one-third of all pituitary adenomas and 14–20% of all clinically relevant adenomas [1,3]. Adenomas under 1 cm in diameter are classified as microadenomas. Non-functioning microadenomas that do not impinge on the optic chiasm are often managed conservatively with observation, as only 14.5% have been shown to grow over five years [4]. Intervention is indicated for NFPAs with a diameter > 1 cm or associated with mass effect symptoms, such as headache, visual compromise, or pituitary apoplexy. First-line treatment is transsphenoidal surgery (TSS), which achieves gross total resection in 19% to 83% of patients, depending on the extent of tumor invasion and other adenoma characteristics [5]. TSS for giant NFPA, defined as those > 4 cm in diameter, achieves gross total resection in 50% of cases [6]. Tumors that invade the cavernous sinus and encase the cavernous carotid artery are difficult to resect fully [7], with gross total resection achieved in 82.6% of Knosp grade 0 tumors compared to 5.9% of Knosp grade 4 tumors [8].

After initial surgical resection, relapse can be defined as the recurrence of a completely resected tumor, with no visible remnant at the first postoperative imaging or as the regrowth of a tumor remnant by more than 2 mm in diameter. In a retrospective study of 142 patients with macroNFPA, with a mean follow-up time of 6.9 years, 24% of patients who achieved gross total resection experienced recurrence, while 47% of patients with a tumor remnant showed tumor growth. The overall relapse rates after surgical resection were 25% at 5 years, 44% at 10 years, and 64% at 15 years [9].

For a patient with an unresectable tumor or clinically significant recurrence, radiation therapy, delivered as either fractionated radiotherapy or stereotactic radiosurgery, is often the next step in treatment. A recent meta-analysis evaluating more than 3000 patients with macroNFPA, who were followed for a mean of 60 months, confirmed that stereotactic radiosurgery achieved local tumor control in 92.3% of patients [10]. Similar tumor control rates were observed in a retrospective cohort study of 45 patients with NFPA treated with hypofractionated radiotherapy [11].

Although surgical resection and radiotherapy control tumor growth in a majority of cases, a subset of NFPAs require further treatment [12]. These tumors are defined by the European Society of Endocrinology (ESE) as aggressive, with the occurrence of radiological invasiveness, a rapid tumor growth rate, or clinically relevant tumor growth, despite optimal standard treatment being administered [13]. Currently, there are no approved medical therapies for aggressive NFPA. However, a number of published studies and case reports have demonstrated the efficacy of various medical therapies in treating NFPAs, including the chemotherapeutic agent temozolomide [13]; somatostatin receptor ligands (SRL), dopamine agonists, immune checkpoint inhibitors, vascular endothelial growth factor (VEGF) inhibitors, and peptide receptor radionuclide therapy. This narrative review considers the available evidence for the use of these therapies in patients with aggressive NFPA and suggests topics for future research.

### 1.1. Temozolomide (TMZ)

First developed in the 1980s, TMZ is an oral alkylating chemotherapy agent that has excellent blood–brain barrier penetration, allowing it to target central nervous system tumors. By methylating DNA guanine bases, the drug creates breaks in DNA that, if not repaired, lead to cellular apoptosis [14,15]. TMZ is approved for the treatment of malignant brain tumors, such as glioblastoma and anaplastic astrocytoma, and has also shown promising results in treating aggressive pituitary tumors. The first recorded use in a pituitary tumor was in 2006, when an elderly male with recurrent prolactin-secreting pituitary carcinoma achieved stable prolactin levels and a significant reduction in the size of the primary tumor and metastases within 18 months of TMZ therapy [16].

The ESE guidelines recommend the use of TMZ in patients with an invasive, fast-growing, or recurrent pituitary tumor, after surgery and radiotherapy [13]. A retrospective study of 27 patients with pituitary adenomas and carcinomas demonstrated >30% tumor volume reduction in 8 patients, tumor stabilization in 14, and tumor progression in 5, after a mean of 13 TMZ cycles. Patients who received concomitant radiotherapy along with TMZ demonstrated significantly higher progression-free survival rates, although the overall survival rates were similar to those treated with TMZ monotherapy [17] (Table 1).

The ESE guidelines suggest the completion of three cycles of TMZ treatment before imaging assessment of the tumor response. If tumor progression is evident, TMZ should be discontinued and alternate systemic therapies should be considered. If a clinical response is seen after three cycles, TMZ treatment should be continued for at least a total duration of 6 months, and possibly longer if a continued therapeutic benefit is seen [13]. Indeed, in a systematic review assessing the effects of long-term TMZ treatment, patients who received >12 months of TMZ had a 5-year progression-free survival rate of 61.3% and 5-year overall survival rate of 91.7% compared to 16.3% and 54.1%, respectively, in those who received <12 months of therapy [18]. Rechallenging with another three cycles of TMZ can also be considered for patients who showed an initial response and then relapsed after completing treatment [13].

Of note, several studies have found TMZ to be less effective in the treatment of NFPAs than in functioning tumors [19,20,21]. In the above-mentioned retrospective study of 27 aggressive tumors, fewer patients with NFPA showed partial responses and more showed progression than those with functioning tumors [17]. A review of 100 reported cases found that 22% of NFPAs responded to TMZ compared to 50% of prolactinomas and corticotroph adenomas [19], and a survey of ESE members found that of 125 aggressive pituitary adenomas treated, 17% of NFPAs demonstrated regression in response to TMZ therapy compared to 45% of functioning tumors [20].

The response to TMZ may be impacted by the tumoral expression of O6-methylguanine-DNA-methyltransferase (MGMT), which repairs the DNA damage caused by alkylating chemotherapy. While this DNA repair mechanism is beneficial for healthy cells, the high expression of MGMT leads to TMZ resistance [15,18,20,22]. In the ESE survey, 76% of tumors with high MGMT expression (>50% of cells) showed no response to TMZ, whereas 46% of tumors with low MGMT expression (<10% positive cells) achieved regression. In addition, six tumors with low MGMT expression exhibited a complete response to TMZ [20]. The aforementioned systematic review comparing long- and short-term TMZ treatment identified a significant association between the negative expression of MGMT and a complete or partial response to TMZ in atypical, aggressive pituitary adenomas [18]. Other biomarkers, such as Ki-67, have also been investigated in regard to their role in the TMZ response, but their impact is not as consistent as that of MGMT [17,18,19].

Some studies suggest a benefit from the empiric use of TMZ prior to the manifestation of clinically aggressive behavior, such as evidence of radiologic invasiveness and elevated proliferative indices during histological assessments [23]. Others suggest that patients with giant adenomas, who require multiple surgical resections, could be considered for TMZ therapy to induce tumor shrinkage prior to initial surgery or after partial resection [24].

TMZ is generally well tolerated; the most common side effects are nausea and vomiting. Seizure and thrombocytopenia are rare side effects, seen in fewer than 7% of patients. As with other alkylating agents, there is a long-term risk of developing myelodysplastic syndrome or acute myeloid leukemia [14,25].

### 1.2. Somatostatin Receptor Ligands (SRLs)

Three SRLs, which bind one or more somatostatin receptors (SST), are approved for use in patients with pituitary adenomas: octreotide, lanreotide, and pasireotide for acromegaly, and pasireotide for Cushing’s disease [26]. Octreotide and lanreotide primarily bind SST2, while pasireotide binds SST1-3 and, specifically, 5 with high affinity [26,27]. While some have found high expression of SST3 and SST5 in NFPA [28,29], the in vitro [30,31] and clinical response to available SRLs have been inconsistent [29,32,33].

In a prospective case-control study of 39 patients with postoperative residual NFPA, those with uptake on SST scintigraphy were treated with long-acting octreotide, while those with negative uptake were assigned to the control group. After a mean follow-up of 37 months, 81% of the treated group had achieved tumor stabilization compared to 47% of the untreated control group [29] (Table 1).

However, in the GALANT randomized control trial of 44 patients with NFPA and SST expression on 68Ga-DOTATATE PET/CT, no significant difference in the tumor diameter was seen between those randomized in regard to 72 weeks of lanreotide versus the placebo [33].

The main adverse effects of SRLs are gastrointestinal distress, including cramping, change in bowel habits, nausea, and, more rarely, biliary tract disease and exocrine pancreatic insufficiency [34].

### 1.3. Dopamine Agonists (DAs)

Dopamine receptors (DR) are expressed in most pituitary adenomas, both functioning and non-functioning. DAs have the highest efficacy in regard to prolactinomas, followed by acromegaly, Cushing disease, and NFPAs [35,36]. NFPAs have been shown to express D2R, and DAs bind with high affinity to D2R [36,37,38]. However, the expression of D2R in NFPA has not been shown to predict the response to DA therapy in vitro or clinically [39,40,41].

Nevertheless, DAs have shown clinical efficacy in the treatment of NFPA (Table 1). Overall, pooled studies have demonstrated that bromocriptine induces the stabilization of NFPA growth in more than 90% of cases [36] and several studies have shown that cabergoline is effective in decreasing and/or stabilizing the tumor size.

In a randomized clinical trial of 144 patients with residual NFPA after surgical resection, 28.8% of those treated with 2 years of cabergoline exhibited a tumor size reduction, while 66.1% achieved stabilization, and 5.1% had continued growth of the tumor remnant. By contrast, those randomized in regard to clinical observations showed a tumor size reduction in only 10.5% of patients, with 73.7% showing stabilization and 15.8% continued tumor growth [39].

A retrospective study of 32 patients treated with adjuvant cabergoline for a visible tumor remnant after surgical NFPA resection, and 12 patients treated with cabergoline as the primary therapy, evaluated the tumor size for a median duration of 30 months. Overall, 66% of the participants achieved >20% reduction in the tumoral size [42]. Similarly, in a retrospective study of 25 surgery-naive patients with NFPA, after 24 months of cabergoline, 20% of the adenomas had decreased in size, 48% remained stable, and 32% had increased in size [43]. Of note, as patients may show a tumor reduction then subsequently experience tumor regrowth [42], the initial response does not necessarily guarantee a long-term response.

In a study of 55 patients with residual NFPA after surgery who were treated prophylactically with cabergoline or bromocriptine, 87.3% had a stable or decreased size of their tumor remnant [40]. In a systematic review of five studies evaluating 187 patients with NFPA, treatment with cabergoline prevented tumor progression after surgical resection in 50% of patients and involved a follow-up over 6 months to over 6 years [44]. In sum, these results suggest DA might be a good option for patients with residual NFPA tumors [45].

The adverse effects of DA include dizziness, nausea, headache, vivid dreams, and impulse control disorders [44,46]. However, these effects are relatively uncommon with doses used in the treatment of pituitary adenomas; one meta-analysis found that only 2% of NFPA patients experienced any adverse event while taking cabergoline. Physical symptoms can generally be controlled with a dose reduction, and impulse control disorders are reversible after drug cessation [44].

Of note, there are pre-clinical and clinical studies of combination therapy involving DA and SRL to treat NFPA [47,48,49,50]; however, there is currently no evidence to suggest that combination therapy is more effective than monotherapy.

### 1.4. Immune Checkpoint Inhibitors (ICIs)

ICIs act by inhibiting various immune checkpoints and, thereby, encourage a stronger anti-tumor response [51]. ICIs that block cytotoxic T lymphocyte-associated protein 4 (CTLA-4) or programmed cell death 1 (PD-1) or its ligand PD-L1 are approved for the treatment of a variety of cancers, including melanoma, non-small cell lung carcinoma, and cervical cancer [52].

PD-1, PD-L1, and CTLA-4 expression have been demonstrated in pituitary adenomas to varying degrees. One study of 139 pituitary tumors, of which 60% were non-functioning, found that 18% of tumors were PD-L1 positive, with no significant difference between functioning and non-functioning tumors [53]. Another study of 115 pituitary adenomas, about half of which were considered invasive, found that functioning pituitary tumors had a significantly higher expression of PD-1 and PD-L1 compared to NFPAs; no differences were found in CTLA-4 expression among tumor subtypes [54]. A third study of 191 pituitary tumors found 58.8% of functioning adenomas had positive PD-L1 immunostaining, compared to 34.2% of NFPAs [55].

Several studies have investigated the response of functioning pituitary tumors to ICIs [51], mostly described in case reports and case series (Table 1). For example, one case report described a 35-year-old woman with an aggressive ACTH-secreting pituitary adenoma, who showed disease progression despite treatment with temozolomide and capecitabine, and developed liver metastases. Treatment with nivolumab and ipilimumab was initiated, and the patient experienced regression of both liver metastases and the primary pituitary tumor volume [56].

An ESE survey of 171 patients with aggressive pituitary tumors and carcinomas identified six patients, five with ACTH-secreting and one with a silent prolactin-staining adenoma, who were treated with either dual or single agent ICIs for 2 to 14 months as second-line therapy. Four patients had progressive disease despite ICI therapy, one showed a transient response to therapy for 3 months before progression, and one had a partial response for 8 months, followed by progression [57].

The one prospective study of ICIs in patients with aggressive pituitary tumors (five corticotroph, four lactotroph, and one somatotroph) found stable disease as the best response in six patients and tumor shrinkage in only two patients [58].

The outcomes of ICI treatment in four NFPAs were reviewed in a series of 13 patients with aggressive pituitary tumors and 16 patients with carcinomas treated with PD-1 inhibitors as a monotherapy or in conjunction with other ICIs. All the patients had previously undergone 1–4 surgeries, as well as various combinations of medical and radiation therapy. In regard to the cohort, eight showed a positive radiological response to PD-1 inhibitor therapy, defined as a complete response, partial response, or stable disease. Among the four patients with NFPAs, the one non-metastatic NFPA showed stable disease following PD-1 inhibitor therapy, while two of the metastatic NFPAs showed a partial response and one showed stable disease [59].

In studies of ICIs in patients with cancer, 54% to 76% of patients experience immune-related adverse events (irAEs), including hypophysitis, thyroid dysfunction, and type 1 diabetes mellitus. PD-1 and PD-L1 inhibitors tend to be better tolerated than CTLA-4 inhibitors, with grade 3 or 4 irAEs making up a larger percentage of irAEs in regard to CTLA-4 inhibitors than PD-1/PD-L1 inhibitors [60].

Two clinical trials are currently studying the effects of nivolumab and ipilimumab on aggressive pituitary adenomas and carcinomas [61,62]. The results of these and other studies will help to develop a better understanding of the role of ICIs in the treatment of aggressive NFPAs.

### 1.5. VEGF Inhibitors

Most pituitary adenomas and carcinomas of all subtypes express moderate-to-high levels of VEGF, and their expression is higher in NFPAs with cavernous sinus invasion compared with those without invasion [63]. The protein increases vascular permeability [64] and promotes NFPA cell viability [41].

The VEGF inhibitor, bevacizumab, was initially approved for use in metastatic colon cancer in combination with chemotherapy. Since then, the addition of bevacizumab to chemotherapy and immunotherapy regimens has increased the overall and progression-free survival in many advanced cancer patients [65].

The data on bevacizumab treatment in patients with pituitary adenomas are limited to case reports and case series (Table 1). In one report, a 44-year-old man with an aggressive silent corticotroph adenoma developed recurrent sellar tumor growth and vertebral metastases, despite multiple surgeries, radiotherapy, and temozolomide. He was treated with bevacizumab for 26 months and achieved stable disease, with no new metastases [66]. In another report, a 63-year-old man with an ACTH-secreting pituitary carcinoma was treated with a combination of radiotherapy, temozolomide, and bevacizumab 6 weeks after surgical resection; 5 years later, he was alive and had no recurrence of his tumor [67].

The ESE survey identified 11 patients with aggressive pituitary tumors or carcinomas who were treated with bevacizumab. One patient achieved a partial response, three achieved stable disease, and five had progression; the response in the remaining two patients was not assessed. The one patient with an NFPA demonstrated stable disease for 7.5 months after treatment with bevacizumab [57].

VEGF inhibitors are generally well tolerated; the most common adverse effects of hypertension, fatigue, asthenia, abdominal pain, and diarrhea can mostly be managed symptomatically [68].

### 1.6. Peptide Receptor Radionuclide Therapy (PRRT)

PRRT can be used in tumors that show elevated levels of SST expression on octreotide scintigraphy or DOTATATE PET/CT [45,69], which may not be readily available in most treatment centers. Few studies of PRRT in patients with aggressive pituitary adenomas have been published [70]. To date, there are no clinical trials of PRRT in regard to NFPA, although a few case reports and case series suggest some efficacy in this treatment approach (Table 1).

One case report described a 55-year-old patient with NFPA who underwent surgery and radiosurgery. After 8 years of stable disease, he developed significant growth of a residual tumor and an oculomotor nerve palsy. An octreotide scintigraphy was positive, and he began PRRT with ^177^Lu-DOTATATE. After three doses, the oculomotor nerve palsy improved, and the patient again showed stable disease for 8 years [71].

Another report described a 63-year-old man with a non-functioning pituitary carcinoma who had disease progression despite several surgeries and radiation therapy. After verifying positive SST expression on DOTATATE PET/CT, he received four cycles of ^177^Lu-DOTATATE therapy. At 40 months, his disease was stable, and he was asymptomatic [70].

The ESE survey identified 11 aggressive pituitary adenomas or carcinomas, four of which were clinically silent, that were treated with PRRT after temozolomide, radiotherapy, and/or bevacizumab. PRRT induced partial regression in three patients, stable disease in two, and progressive disease in the remaining six. Two of the four clinically silent tumors had a partial response and two had stable disease [57].

PRRT induced stable or decreased the tumor burden, as well as clinical or biochemical improvements in 4 of 13 patients with functioning or non-functioning aggressive or metastatic pituitary tumors. Of note, the response to PRRT did not correlate with patient age or sex, but resistance to PRRT was significantly associated with prior treatment with temozolomide. That is, every patient who was previously treated with temozolomide did not show a response to PRRT [72].

Most patients in these reports did not experience any adverse effects from PRRT. One patient had a transient thrombocytopenia and one had severe facial pain after treatment [70,72]. Renal failure and bone marrow toxicity have also been reported [71,72].

**Table 1:** Summary of published evidence on the treatment of aggressive NFPAs.

	N *	Efficacy/Response Rate in NFPA	Predictors of Response	Source(s)
TMZ	82 NFPAs	17–79% tumor stabilization or >30% tumor size reduction	Low MGMT expression (+), concomitant radiotherapy (+), longer treatment duration (+), non-functioning (−) vs. functioning (+)	CR, clinical studies, in vitro studies
SRL	182 NFPAs	Mixed results: some show 80% tumor stabilization or reduction; others no impact	High uptake on SST scintigraphy (+)	CR, case-control study, in vitro studies
DA	455 NFPAs	20–87% tumor size reduction, 48–66% tumor stabilization	Unknown	Clinical trials, CR, CS
ICI	38 adenomas/carcinomas (7 NFPAs)	28–60% stable disease, partial response, or complete response	High PD-1, PD-L1, and CTLA-4 expression (+)	CR, CS
VEGF inhibitors	30 adenomas/carcinomas (4 NFPAs)	44% stable disease or partial response	Unknown	CR, CS
PRRT	32 adenomas/carcinomas (9 NFPAs)	31–46% stable disease or partial response	High expression of SST (+), prior TMZ (−)	CR, CS

* Single-case reports not included; (+), positive; (−), negative; CR, case report; CS, case series; CTLA-4, cytotoxic T-lymphocyte associated protein 4; DA, dopamine agonist; ICI, immune checkpoint inhibitor; MGMT, O-6-methylguanine-DNA methyltransferase; NFPA, non-functioning pituitary adenoma; PD-1, programmed cell death 1; PD-L1, programmed cell death ligand 1; PRRT, peptide radionuclide therapy; SRL, somatostatin receptor ligand; SST, somatostatin receptor; TMZ, temozolomide; VEGF, vascular endothelial growth factor. References: [13,15,16,17,18,19,20,21,22,29,33,39,40,41,42,43,51,53,54,55,56,57,58,66,67,70,71,72].

## 2. Methods

A literature search was carried out on PubMed and Google Scholar using the search terms nonfunctioning pituitary adenoma, medical therapy, temozolomide, somatostatin receptor ligand, octreotide, lanreotide, pasireotide, dopamine agonist, bromocriptine, cabergoline, immune checkpoint inhibitor, nivolumab, ipilimumab, vascular endothelial growth factor inhibitor, bevacizumab, peptide receptor radionuclide therapy. Inclusion criteria included papers published in reputable journals between the years 2000 to 2025, with preference given to more recently published papers and papers published in English. Certain publications were excluded if they contained data or case studies that were already accounted for. Preference was given to studies that included clinical data, however select in-vitro studies were included if they provided insight on predicted response to therapy or if minimal clinical data was available for a treatment.

## 3. Discussion

The absence of approved therapies for aggressive NFPAs, the limited data available from clinical trials, and the lack of biochemical markers of response all contribute to the challenge of treating these tumors. Of the therapies reviewed here, the benefit from the use of TMZ, SRL, and DA in NFPA is most well-documented, while the potential benefit of ICIs, VEGF inhibitors, and PRRT is largely unknown.

The ESE guidelines recommend TMZ as first-line chemotherapy for the treatment of pituitary tumors that are invasive, rapidly growing, and recur despite conventional therapies [13]. However, several studies show that only a minority of NFPAs exhibit a tumoral response to TMZ compared to functioning adenomas. Molecular testing of a tumor after surgery for MGMT expression could be helpful for identifying potential responders to TMZ. Yet, the timing of when to initiate TMZ is unclear, with some suggesting earlier treatment post-surgery may be warranted in patients whose tumors show invasive or aggressive features [23]. Temozolomide has shown greater efficacy when used in combination with radiotherapy, but further studies are needed to verify a clinically significant effect on NFPAs.

Of the three SRLs in clinical use, only octreotide has shown clinical efficacy in inducing NFPA stabilization. Although NFPAs express SST3 and SST5, pasireotide, which has an affinity for these receptors, is not particularly effective in the treatment of NFPAs.

DAs are the most well-studied medical therapy for aggressive NFPAs. The results of a randomized control trial, retrospective studies, and a systematic review have shown that more than 2/3 of patients with NFPA show tumor stabilization or shrinkage following DA treatment. Similar to what is seen with the SRL response and SST expression, cabergoline and bromocriptine have a high affinity for D2R, yet NFPA expression of D2R does not correlate with the treatment response. Nonetheless, given the consistent results of clinical studies in patients with NFPA and the relative safety of DA, clinicians may choose to use these agents empirically in patients with incompletely resected tumors.

ICIs are being evaluated in functioning pituitary adenomas, with a modest clinical response. Few NFPAs have been included in these studies and the expression of PD-1 and PD-L1 is lower in these tumors. The clinical trials of ICI therapy that are currently underway are expected to provide further insight into ICIs as a potential treatment for aggressive NFPAs.

VEGF expression is higher in NFPAs with cavernous sinus invasion, yet clinical evidence for its use in this setting is sparse. The few cases reported show the efficacy of bevacizumab in stabilizing and preventing the recurrence of non-functioning pituitary carcinoma and ACTH-secreting pituitary carcinoma. However, there are currently no clinical trials investigating VEGF inhibitor use in the treatment of aggressive pituitary adenomas.

The efficacy of PRRT in the treatment of NFPA is also limited to case reports, and, so far, they show mixed results. One review of published cases found that patients previously treated with TMZ had a particularly poor response to PRRT, suggesting that the timing of the treatment may be an important consideration. Large clinical studies of PRRT in pituitary adenomas, and specifically NFPAs, are needed to determine the treatment’s efficacy and safety. However, as most providers are not likely to have access to a method that quantifies SST expression, it will likely be difficult for them to offer PRRT to their patients.

A personalized approach may prove most helpful in selecting a medical therapy that is most beneficial for a patient with an aggressive NFPA. Recent updated guidelines from the ESE suggest molecular analysis for the genes *TP53* and *SF3B1* in lactotroph tumors and *TP53* and *ATRX* in corticotroph macroadenomas [73]. Certain studies have reported pathogenic variants of these genes in higher numbers in adenomas that behave aggressively. These analyses may help to predict aggressive behavior in tumors, although there are not yet targeted therapies for the genes *TP53*, *SF3B1*, and *ATRX* [71]. Pending additional studies confirming their value in NFPA, measuring expression of MGMT, somatostatin and dopamine receptors, PD-1/PD-L1, CTLA-4, and VEGF can be done routinely on resected tissue in patients with aggressive tumors. As noted by the ESE, this may be particularly useful for tumors that have already been shown to be resistant to TMZ, as results can guide clinicians in choosing a subsequent targeted therapy most likely to be effective [73].

A multidisciplinary team approach is critical when selecting treatment for patients with aggressive NFPAs. Endocrinologists, radiation oncologists, neurosurgeons, pathologists, radiologists, and primary care doctors can all contribute to the decision-making process, defining the patient’s goals and determining the balance between maximizing treatment benefits and minimizing the burden of care.

## 4. Future Directions

1. Many published studies on the medical treatment of aggressive pituitary tumors group together functioning and non-functioning tumors in the analysis. Providing results by pathology type would allow clinicians to better discern the effect of each therapy on aggressive NFPAs.
2. The available data are mostly derived from observational studies. Randomized controlled clinical trials are needed to compare the efficacy and safety of different treatment modalities.
3. More in-depth studies using next-generation sequencing and histologic analysis on specimens from patients with aggressive tumors will help to determine whether the expression of somatostatin and dopamine receptors, MGMT, VEGF, PD-1/PD-L1, and CTLA-4, and other markers can be used to personalize treatment selection.

## Data Availability

No new data were created.
